# Malaria indicator survey 2007, Ethiopia: coverage and use of major malaria prevention and control interventions

**DOI:** 10.1186/1475-2875-9-58

**Published:** 2010-02-24

**Authors:** Daddi Jima, Asefaw Getachew, Hana Bilak, Richard W Steketee, Paul M Emerson, Patricia M Graves, Teshome Gebre, Richard Reithinger, Jimee Hwang, the Ethiopia Malaria Indicator Survey Working Group

**Affiliations:** 1 Diseases Prevention and Control Department, Federal Ministry of Health, Addis Ababa, Ethiopia; 2 Malaria Control and Evaluation Partnership in Africa (MACEPA), a programme at PATH, Addis Ababa, Ethiopia; 3 Malaria Control and Evaluation Partnership in Africa (MACEPA), a programme at PATH, Ferney-Voltaire, France; 4 The Carter Center, Atlanta, USA; 5 The Carter Center, Addis Ababa, Ethiopia; 6 US Agency for International Development, Addis Ababa, Ethiopia; 7 US Centers for Disease Control and Prevention, Atlanta, USA; 8 Global Health Group, UCSF Global Health Sciences, San Francisco, USA

## Abstract

**Background:**

In 2005, a nationwide survey estimated that 6.5% of households in Ethiopia owned an insecticide-treated net (ITN), 17% of households had been sprayed with insecticide, and 4% of children under five years of age with a fever were taking an anti-malarial drug. Similar to other sub-Saharan African countries scaling-up malaria interventions, the Government of Ethiopia set an ambitious national goal in 2005 to (i) provide 100% ITN coverage in malarious areas, with a mean of two ITNs per household; (ii) to scale-up indoor residual spraying of households with insecticide (IRS) to cover 30% of households targeted for IRS; and (iii) scale-up the provision of case management with rapid diagnostic tests (RDTs) and artemisinin-based combination therapy (ACT), particularly at the peripheral level.

**Methods:**

A nationally representative malaria indicator survey (MIS) was conducted in Ethiopia between September and December 2007 to determine parasite and anaemia prevalence in the population at risk and to assess coverage, use and access to scaled-up malaria prevention and control interventions. The survey used a two-stage random cluster sample of 7,621 households in 319 census enumeration areas. A total of 32,380 people participated in the survey. Data was collected using standardized Roll Back Malaria Monitoring and Evaluation Reference Group MIS household and women's questionnaires, which were adapted to the local context.

**Results:**

Data presented is for households in malarious areas, which according to the Ethiopian Federal Ministry of Health are defined as being located <2,000 m altitude. Of 5,083 surveyed households, 3,282 (65.6%) owned at least one ITN. In ITN-owning households, 53.2% of all persons had slept under an ITN the prior night, including 1,564/2,496 (60.1%) children <5 years of age, 1,891/3,009 (60.9%) of women 15 - 49 years of age, and 166/266 (65.7%) of pregnant women. Overall, 906 (20.0%) households reported to have had IRS in the past 12 months. Of 747 children with reported fever in the two weeks preceding the survey, 131 (16.3%) sought medical attention within 24 hours. Of those with fever, 86 (11.9%) took an anti-malarial drug and 41 (4.7%) took it within 24 hours of fever onset. Among 7,167 surveyed individuals of all ages, parasitaemia as estimated by microscopy was 1.0% (95% CI 0.5 - 1.5), with 0.7% and 0.3% due to *Plasmodium falciparum *and *Plasmodium vivax*, respectively. Moderate-severe anaemia (haemoglobin <8 g/dl) was observed in 239/3,366 (6.6%, 95% CI 4.9-8.3) children <5 years of age.

**Conclusions:**

Since mid-2005, the Ethiopian National Malaria Control Programme has considerably scaled-up its malaria prevention and control interventions, demonstrating the impact of strong political will and a committed partnership. The MIS showed, however, that besides sustaining and expanding malaria intervention coverage, efforts will have to be made to increase intervention access and use. With ongoing efforts to sustain and expand malaria intervention coverage, to increase intervention access and use, and with strong involvement of the community, Ethiopia expects to achieve its targets in terms of coverage and uptake of interventions in the coming years and move towards eliminating malaria.

## Background

Approximately 75% of Ethiopia's landmass is endemic for malaria, with malaria primarily associated with altitude and rainfall [1-3]. In general, the peak of malaria incidence follows the main rainfall season (July - September) each year. However, many areas in the south and west of the country have a rainfall season beginning earlier in April and May or have no clearly defined rainfall season [4]. Depending on these rainfall patterns, transmission tends to be highly heterogenous geo-spatially within each year as well as between years. Additionally, malaria in Ethiopia is characterized by widespread epidemics occurring every 5-8 years, with the most recent one between 2003 and 2005 [5,6]. In 2007/2008, malaria was the first cause of outpatient visits, health facility admissions and in-patient deaths, accounting for 12% of out-patient visits and 9.9% of admissions [7].

Demographic and Health Surveys (DHS) were carried out in Ethiopia in 2000 and 2005, and included a malaria module [8,9]. Recognizing the low coverage and use of malaria interventions in the country, in 2005 the Government of Ethiopia's Federal Ministry of Health (FMOH) developed a 5-year National Malaria Prevention and Control Strategy [10]. According to the strategy, areas <2,000 m were considered 'malarious' and targeted to receive key malaria control interventions, including insecticide-treated nets (ITNs), indoor residual spraying of households with insecticide (IRS), and rapid diagnostic tests (RDTs) for malaria coupled with prompt and effective case management with artemisinin-based combination therapy (ACT). The strategy outlined an ambitious national goal of 100% household ITN coverage in malarious areas with a mean of two LLINs per household through distribution of about 20 million LLINs by the end of 2007. Moreover, the strategy stated that IRS should be scaled-up to cover 30% of households targeted for IRS and also included the rapid scale-up of provision of RDTs and ACT to newly established community health posts.

Implementation of the above strategy greatly benefited from two grants from the Global Fund to Fight AIDS, Tuberculosis and Malaria (GFATM): Round 2 (2002 - 2008; total budget: $73 million) and Round 5 (2005 - 2010; total budget: $140 million) [11]. Recently, Ethiopia was successful in applying for Round 8 funding support (2008 - 2013; $276 million). With the above support as well as the support from other in-country malaria stakeholders, according to FMOH records between 2004 and 2007 a total of 12.5 million RDTs, 15.4 million ACT treatment courses and 17.2 million ITNs were distributed to malarious areas of the country. Additionally, the number of structures targeted for IRS increased from 3.4 to 4.2 million [12].

Malaria Indicator Surveys (MISs) were developed by the Roll Back Malaria (RBM) Monitoring and Evaluation Reference Group (MERG) with the aim to help national Ministries of Health collect key and timely information on malaria control at the national level [13]. Additionally, the information collected during MISs is comparable with existing DHS and multiple indicator cluster surveys (MICS) protocols, which allows for comparison of data amongst the surveys and monitors the progress of National Malaria Control Programme efforts.

To assess the coverage and impact of scaled-up malaria interventions, the FMOH decided to conduct a MIS in 2007. Specific objectives of the MIS in Ethiopia were (i) to evaluate access, coverage and use of key malaria prevention and control interventions; (ii) to obtain nationally representative data to assess parasitaemia and anaemia in populations at risk of malaria; and (iii) to assess the FMOH's progress towards national and global malaria goals.

## Methods

### Study approach

The MIS was conducted from October through December 2007. The protocol for the MIS followed RBM MERG guidelines [13] with a few local modifications [14]. To generate nationally representative data, a stratified two-stage cluster sample design with census enumeration areas (EAs; comprising approximately 200 households) as primary sampling units was used, stratified by several domains, including altitude (i.e. <1,500 m *vs*. 1,500 - 2,500 m) and degree of urbanization (i.e. rural *vs*. urban).

For Amhara and Oromia Regional States, there was over-sampling of EAs so that samples for estimating malaria indicators at regional state level could be generated. This was done to accommodate the needs of The Carter Center and the President's Malaria Initiative, who required regional-level indicator data to monitor implementation and impact of their respective programmes. The Carter Center is implementing comprehensive trachoma, onchocerciasis and malaria programme activities in Amhara Regional State [15], and Oromia is the focus regional state for the President's Malaria Initiative [16].

### Sample size determination and allocation

The sample size was determined using 95% confidence limits, 80% power, a design effect of 1.25, and 20% adjustment for non-response (i.e. from household refusals or abandoned households). In addition, the sample size assumed that 82% of households had children <6 years of age. Based on the above inputs and assumptions, a minimum sample of 5,650 households was determined to be necessary to obtain robust national level information for altitude and urbanization categories. An additional 2,875 households were included in order to get regional state estimates for Amhara and Oromia. Consequently, the total sample size of the survey was estimated to be 8,525 households.

Taking into account sample precision, logistics and survey cost, it was decided that a randomly selected sample of 25 households per EA would be optimum; five households per EA could be additionally selected to compensate for absentee or abandoned households.

### Sampling weights and estimation procedures

Because of oversampling in some domains (e.g. in Oromia and Amhara) and because the number of sampled households in each EA was fixed, the sample was not self-weighting (i.e. each EA and each household did not have equal probability of selection). Therefore, weights were used to compensate for the resulting differential selection probabilities. Sampling weights were computed based on the implemented survey design as the inverse of the product of the sampling probability and indicator estimates were calculated using those weights.

### Survey organization and data collection

In each selected EA, all households were mapped, and 25 and five alternate households were randomly selected by personal digital assistants (PDAs) (Hewlett -Packard IPAQ HX249X, Palo Alto, CA, and Dell Axim-51, Round Rock, TX) equipped with global positioning systems (GPS). Interviews regarding malaria indicators were conducted in selected households. The MIS questionnaires, household listing, sampling framework, and navigation programmes were directly programmed into the PDAs using Windows Mobile 5.0 (Microsoft Corporation, Seattle) to allow for paper-free data collection. The programme on the PDAs enabled surveyors to enter second stage sampling (i.e. household listing within an EA, random selection of 25 households, household members) and navigate to selected households to complete interviewing and specimen collection and testing (see below).

Surveyors were organized in 25 teams, with each survey team consisting of six people: four surveyors, one driver, and one team leader. Upon arrival in a selected community, sub-teams of two surveyors dispersed in different directions to map all the households. Some of the teams (i.e. those assigned to the most remote areas of the country) included one additional laboratory technician to process blood slides in the field. Each team carried a standard lot of supplies and materials, consisting of PDAs with their accessories, a map of selected EAs selected by the Central Statistical Authority, uniforms, reagents and instruments for sample collection, testing, and smear preparation, anti-malarial and anti-helminthic drugs, iron syrup or tablets, sensitization letters, and camping equipment.

Teams were visited by supervisors in the field at least twice during the survey period. The objectives of the supervisory visits were to ensure the quality and quantity of data collected by surveyors. Supervisory visits included the following: 1) inspection of teams' PDA records; 2) random inspection of some households by navigating to and visiting completed households; 3) confirmation from the households of the records obtained from the survey; 4) completion of supervisory checklist by direct and indirect observation; and 5) observing a team's overall harmony and performance as well as providing feedback and sharing the experiences of other teams.

### Survey questionnaires

The questionnaires used included two structured, pre-coded questionnaires with both closed- and open-ended questions: (i) a household questionnaire and (ii) a women's questionnaire. Both were based on RBM MERG MIS Questionnaires [13], modified to local conditions. The questionnaires were translated and printed in Amharic, Afaan Oromoo and Tigrigna languages and field-tested in non-survey EAs to determine the validity of the pre-coded answers.

The household questionnaire was administered to the household head or another adult if the household head was absent or unable to respond for any reason, and collected the following data: socio-demographic information and listing of household members; house construction materials and design; ownership of durable assets; availability, source of origin, type, condition and use of household mosquito net(s) (verified by observation); and reported status of IRS. Additionally, the purpose of the household questionnaire was used to identify children <6 years of age for specimen collection as well as women aged 15 - 49 years who were eligible to answer the women's questionnaire.

The women's questionnaire was administered to women aged 15 - 49 years identified from the household questionnaire and collected the following data: educational level; reproduction, birth history, and current pregnancy status; knowledge, attitudes and practices (KAP) on malaria preventive and curative aspects; reported history of fever among children <5 years of age (U5) in the previous two weeks; and reported treatment seeking behaviour for children U5 with fever.

### Malaria parasite and anaemia testing

Blood samples were taken from all children <6 years of age and from all household members in every fourth household. All children <6 years of age were included to ensure that no children U5 were missed during the survey, and only data for children U5 are presented here. The malaria diagnostic tests included RDTs, blood slides for microscopic examination and haemoglobin level testing. RDTs were used in the survey to offer immediate treatment to individuals with a positive test. The RDT used (ParaScreen^®^, Zephyr Biomedical Systems, India) is a HRP2/pLDH-based antigen test detecting both *Plasmodium falciparum *and other *Plasmodium *spp. (in Ethiopia most likely *Plasmodium vivax*). Sensitivity and specificity of the test in operational conditions in Ethiopia were previously estimated to be 47.5% and 98.5%, respectively [17]. The specimen processing was organized in such a way that all three tests were performed simultaneously from a single finger prick. Two blood slides, thick and thin films (in duplicate), were taken for each participant by a laboratory technician as per standard WHO-approved protocol [18]. Slides were labelled and air-dried horizontally in a carrying case in the field, and stained with Giemsa at the nearest health facility when the team returned from the field usually on the same or the next day. Blood slides were read at a reference laboratory in Addis Ababa and classified qualitatively. One hundred high power fields of the thick film were examined before recording a slide as negative. If positive, the thin film was read to determine the species. To ensure accuracy, all positive slides and a random sample of 5% of the negative slides were re-examined by a second microscopist, who was blinded to the diagnosis of the first slide-reader. The second slide from each participant was used if the first was damaged or unreadable.

An error in the auto-generate function of the PDAs led to a mislabelling of some slides, which subsequently could not be matched to their respective RDT results. Most slides, however, were able to be matched to at least the EA or household levels. For individual level analyses, EAs without 100% slide matches were excluded from the analyses.

Anaemia testing followed the recommendations of the RBM MERG [19], with haemoglobin concentrations measured using a portable spectrophotometer (HemoCue^®^, Anglom, Sweden). The following anaemia classification was used: haemoglobin levels below 11 g/dl, 8 g/dl and 5 g/dl were classified as mild, moderate-severe and severe anaemia, respectively [19].

### Treatment

All individuals surveyed with positive RDTs were offered treatment according to the FMOH's National Diagnosis and Treatment Guidelines [20], i.e. artemether-lumefantrine combination therapy (CoArtem^®^, Novartis, Basel, Switzerland) for *P. falciparum *infection, chloroquine for other *Plasmodium *infections, and referral for clinic-based quinine therapy for self-reported pregnant women.

For children diagnosed with moderate-severe anaemia (i.e. haemoglobin <8 g/dl), results were shared with the parent/guardian and the children were given artemether-lumefantrine (if older than four months) [20], albendazole (if >24 months of age as per National Protocol for Integrated Maternal and Child Illnesses [21]) and a two-week supply of supplemental iron. All infants under four months with a positive RDT result and children with severe anaemia, haemoglobin <5 g/dl, were referred to the nearest health facility for further evaluation and treatment. Subjects who were found to be severely ill, as determined by the survey nurses, were consulted to immediately visit the nearest possible health facility.

### Data management and analysis

Survey data was downloaded from PDAs into a Microsoft ACCESS database (Microsoft Corporation, Seattle). Data management and analysis were carried out in SPSS 16.0 (SPSS, Inc., Chicago, IL), SAS 9.2 (SAS Institute Inc., Cary, NC), and STATA 9.2 (Stata Corporation, College Station, TX). Descriptive statistics were used to describe the characteristics of the sample and calculate coverage, use and access estimates. Point estimates and confidence intervals were derived using the PROC SURVEY (SVY) commands in SAS, which adjusts for clustering in the sampling design, with weighting for household and cluster sampling probability.

### Ethical clearance

The MIS 2007 protocol received ethical clearance from the Emory University Institutional Review Board (IRB# 6389), the U.S. Centers for Disease Control and Prevention Ethical Review Committee (IRB# 990132), the PATH Ethical Committee, and the Ethiopian Science and Technology Agency. Verbal informed consent to participate in interviews was sought from the heads of households and each eligible individual in accordance with the tenets of the Declaration of Helsinki. Verbal informed consent was sought from each eligible individual and parents of children <6 years of age for blood films. Additional verbal informed assent was sought from children aged 6-18 years.

## Results

### Characteristics of sample

Overall, 319 EA consisting of 60,346 households were mapped and 7,621 households were surveyed. Household size ranged from 1 - 26 persons, with a mean of 4.5 (95% C.I. 4.3 - 4.7). Of the 32,380 individuals in the surveyed households, 5,243 (16.7%) were children U5 and 570 (1.7%) were pregnant women. The data presented below are for 'malarious areas' only, defined by the FMOH as areas with an altitude <2,000 m.

### Net ownership

**Figure 1:**
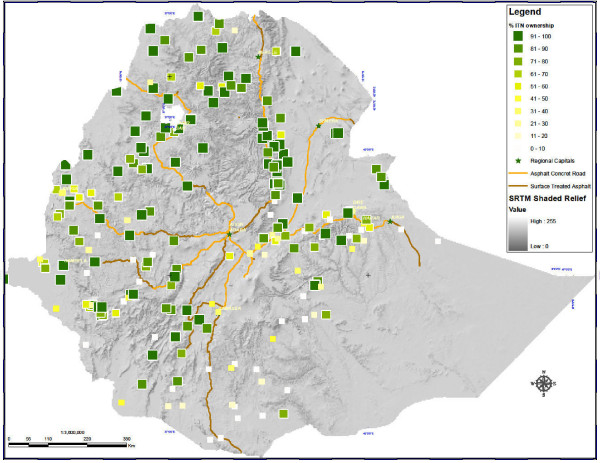
**ITN coverage in Ethiopia: Percentage of households in enumeration areas owning at least one insecticide-treated net**.

Of 5,083 surveyed households in malarious areas, 3,419 (69.0%) owned at least one mosquito net, and 3,282 (65.6%) owned at least one ITN (Figure 1). Of nets surveyed <2,000 m, 95.0% were LLINs. Subsequent analyses refer to ITNs only and are summarized in Table 1.

**Table 1:** Characteristics of net ownership and use for households in clusters <2,000 m

In all HH (N = 5,083)	% owning ≥ 1 ITN		65.6%
	
	% owning ≥ 2 ITN		36.8%
	
	Mean number of ITNs per HH		1.1
Among persons in all HH	% sleeping under ITN last night	All ages	36.1% (N = 21,479)
		
		Children <5 years of age	41.5% (N = 3,643)
		
		Women 15 - 49 years of age	41.7% (N = 4,466)
		
		Pregnant women	42.8% (N = 412)

In HH with ≥ 1 ITN (N = 3,282)	Mean no of ITN per HH		1.7
	
	% of HH where all persons slept under net last night		36.4%
	
	% of HH where at least one ITN was used last night		72.1%

Among persons in HH with ≥ 1 ITN	% sleeping under ITN last night	All ages	53.2% (N = 14,326)
		
		Children <5 years of age	60.1% (N = 2,496)
		
		Women 15 - 49 years of age	60.9% (N = 3,009)
		
		Pregnant women	65.7% (N = 266)

Note, abbreviations: HH, household; ITN, insecticide treated net.

Of ITN-owning households, 56.1% owned more than one ITN, with the mean number of ITNs owned being 1.7 (95% CI 1.6 - 1.9). Since most nets owned were nets distributed free to beneficiaries by the FMOH since 2005, ITNs were primarily blue, family-size (i.e. 180 × 180 × 150 cm) rectangular PermaNet^® ^2.0 (Vestergaard Frandsen, Copenhagen, Denmark); 94.3% of ITNs were reportedly <3 years old.

### Net use

**Figure 2:**
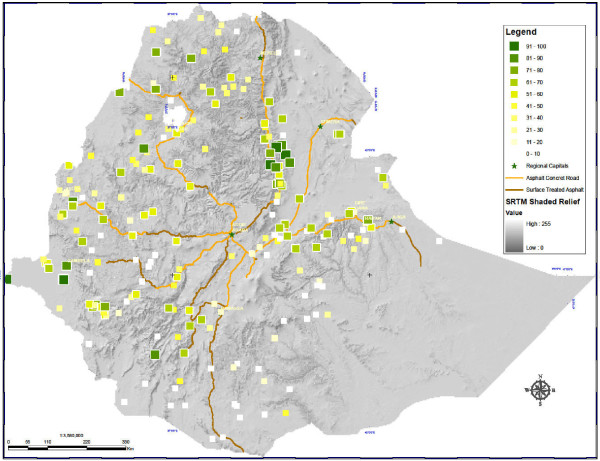
**ITN use in Ethiopia: Percentage of households in enumeration areas using at least one insecticide-treated net**.

Overall, 7,901/21,479 (36.1%) surveyed individuals had slept under an ITN the prior night (Figure 2), including 1,564/3,643 (41.5%) children U5, 1,891/4,466 (41.7%) women 15 - 49 years of age, and 166/412 (42.8%) self-reporting pregnant women.

Within ITN-owning households, 53.2% of all persons had slept under an ITN the prior night, including 1,564/2,496 (60.1%) children U5, 1,891/3,009 (60.9%) women 15 - 49 years of age, and 166/266 (65.7%) pregnant women. In 1,249/3,282 (36.4%) ITN-owning households, all family members reportedly had slept under an ITN the prior night. Among 3,282 ITN-owning households, at least one ITN was used the previous night in 72.1% of households.

### Indoor residual spraying

**Figure 3:**
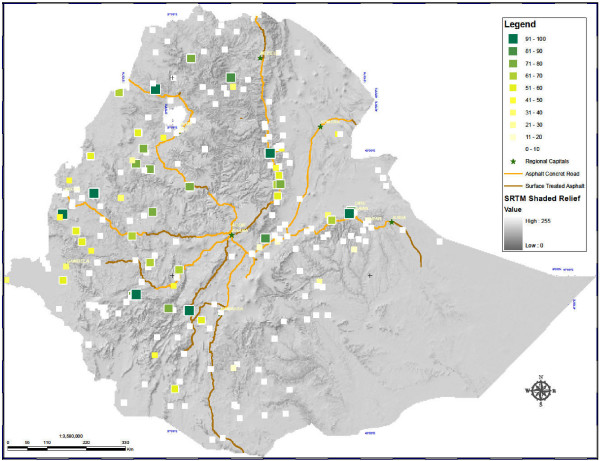
**IRS coverage in Ethiopia: Percentage of households in enumeration areas having been sprayed with residual insecticide in the 12 months preceding the survey**.

Of 5,083 surveyed households, 906 (20.0%) had been sprayed with indoor residual insecticide in the last 12 months (Figure 3). The mean number of months since spraying was 4.7 months (95% C.I. 4.1 - 5.4). Among households sprayed in the last 12 months, 87.6% also had at least one ITN; and among ITN-owning households, 26.7% had been sprayed within the last 12 months.

### Access to treatment

Fever in the previous two weeks for children U5 was reported by the mother for 747/3,041 (24.0%) of children included in the survey. Treatment was sought for 131 (16.3%) of these children within 24 hours of onset of fever and 86 (11.9%) took an anti-malarial drug. Among children who were treated with an anti-malarial drug, 41 (40.0%) took it within 24 hours of onset of fever. Among the febrile children who were treated with an anti-malarial drug within 24 hours of onset of fever, six (10.8%) sought their treatment from a health extension worker, two (2.6%) from other level of government health facility, nine (22.6%) from private health providers, 16 (39.7%) used home treatment, and eight (24.3%) sought treatment from shops.

### Malaria knowledge

Of 4,438 surveyed women, 3,519 (79.5%) had heard of malaria. However, only 2,244 (50.8%) recognized fever as a sign of malaria, 1,763 (41.2%) mentioned mosquito bites as the cause of malaria, and 1,792 (38.2%) cited mosquito nets as a prevention method for malaria.

### Malaria and anaemia prevalence

**Figure 4:**
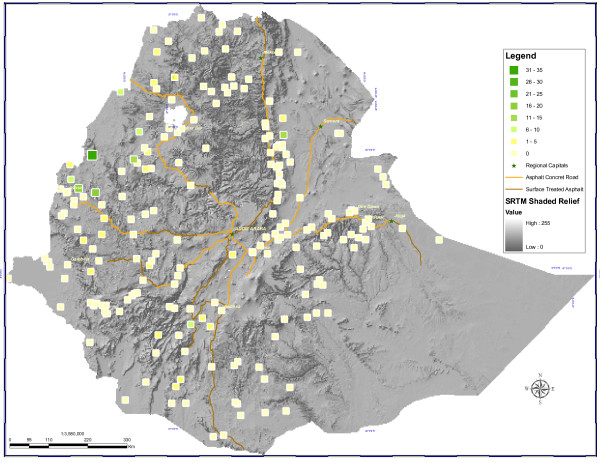
**Distribution of *Plasmodium *infection in Ethiopia: Percentage of surveyed individuals in enumeration areas positive for *Plasmodium falciparum *or *P. vivax *by microscopy examination**.

Overall, 60/7,167 (1.0%) surveyed individuals tested positive for *Plasmodium *infection by microscopy (Figure 4), with 0.7% and 0.3% due to *P. falciparum *and *P. vivax*, respectively. Due to the PDA recording error, 392 blood slides could only be matched to the household level and not to specific individuals surveyed. Of the 6,775 matched individuals, 40 (0.6%) and five (0.1%) tested positive for *P. falciparum *or *P. vivax *using microscopy, respectively; no individuals tested positive for both *P. falciparum *and *P. vivax*. Prevalence of infection in children U5 was 0.9% (26/3,173). Of 45 positive individuals tested, 37 (87.0%) were children <15 years of age. Among children U5, fever was a predictor of parasitaemia. Among the 24% of children with reported recent fever, 86.9% had received no anti-malarial treatment and had blood smear results; and 2.2% of these were parasitaemic - this was statistically different from the parasitaemia rate in children without recent reported fever.

The mean haemoglobin value observed in 3,366 children U5 sampled was 11.0 g/dl (95% C.I. 10.8 - 11.2). Moderate-severe anaemia (<8 g/dl) was observed in 239 (6.6%) of surveyed children U5. Anaemia was significantly associated with blood slide positivity (OR = 3.6; 95% C.I. 1.3-9.8).

Overall, 134/6,815 (2.0%) surveyed individuals tested positive for *Plasmodium *infection by RDTs, with 1.8% and 0.2% due to *P. falciparum *and *P. vivax*, respectively. All positive individuals received on site treatment as per national treatment guidelines [20].

## Discussion

In line with the Global Malaria Action Plan [22] and with the substantial increase in funding support for malaria prevention and control programmes, countries across sub-Saharan Africa are scaling-up national coverage of key malaria prevention and control interventions, including ITNs, IRS, and provision of early diagnosis and treatment.

**Figure 5:**
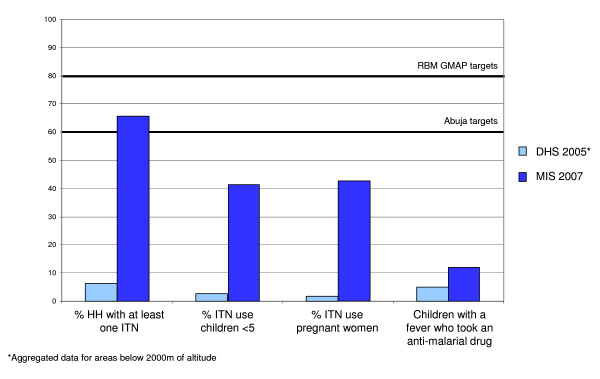
**Progress in key malaria programme indicators 2005 - 2007 compared to Roll Back Malaria Abuja targets**.

As evidenced by the data presented here, Ethiopia has made remarkable progress scaling up these key interventions. In 2000, an estimated 0.2% of households in Ethiopia owned an ITN [8]. Aggregated data from the DHS 2005 showed that in areas <2,000 m, this percentage had risen to 6.4% in 2005 [9] and -as shown here- two years later this percentage has risen to 65.6% (Figure 5). For sub-Saharan African countries with reported national bed net coverage data, this more than 10-fold increase in bed net ownership within two years has catapulted Ethiopia, previously in third to last position in the rankings, to among the countries with highest ITN coverage, along with Togo, Sierra Leone, Zambia and Rwanda [23]. ITN use in all population groups increased significantly from 2000 and 2005 to 53.2% in 2007; similar trends are observed in bed net use in children U5, women 15 - 49 years of age, and pregnant women.

Although some progress in IRS and case management has been made compared to 2000 and 2005, increases in coverage and use of these interventions has been modest. Whereas in 2005, 17.0% of households had been sprayed with insecticide [9], 20.0% had been sprayed in 2007. Similarly, even though case management has improved since 2005, with 11.9% of children U5 with a fever taking an anti-malarial drug in 2007 compared to 4.0% in 2005, it is still below the RBM targets of 60% (Figure 5). Of note, however, is that this particular indicator includes fever cases of non-malarial aetiology, which represent up to 80% of all fever cases, and hence may misrepresent programme performance in terms of access and use of malaria diagnosis and treatment services.

The increase in intervention coverage in Ethiopia is remarkable on several fronts. Between mid-2005 and late 2007, with adequate funding for approximately 20 million ITNs, the programme was able to procure and deliver enough ITNs to achieve a nation-wide 10-fold increase in ownership to approximately 67% of households in malaria-endemic areas. During the same interval, IRS was expanded to target and reach approximately 4.2 million households (a ~25% increase from previous targets), diagnosis with microscopy and RDTs was expanded and ACT was introduced. Ethiopia has demonstrated that with strong leadership and adequate resources, the malaria control programme can rapidly scale-up its full intervention package.

The observed high intervention coverage coincides with low malaria prevalence (i.e. 1.0%), with 70% and 30% of cases due to *P. falciparum *and *P. vivax*, respectively. Observed prevalence is lower than the prevalence observed in previous surveys, including a survey in Amhara, Oromia and SNNP Regional States that had reported a prevalence of 4.1% at the end of 2006 [15,24]. Similarly, child anaemia prevalence was modest and although anaemia was associated with malaria infection in the relatively few parasitaemic children, it is likely that much of the remaining anaemia in children is due to other causes (e.g. nutrition, schistosomiasis, soil-transmitted helminths).

The decrease in malaria prevalence is consistent with findings from other countries that high coverage of malaria control interventions [25] probably contributed importantly to the decrease in population infection rates and, consequently, the threat of potential malaria epidemics. However, attributing the decrease in parasitaemia to the scale-up of key malaria interventions (mainly ITNs and ACTs) should be done with caution. Malaria transmission in Ethiopia is very seasonal and highly variable at micro-geographical scales. Although the MIS was carried out during the country's main malaria transmission season, it was a national cross-sectional survey that only yielded national point prevalence data. Furthermore, it is to be noted that the FMOH carried out a nationwide 'Anti-Malaria Millennium Campaign' just prior to the MIS, which included mass treatment of febrile cases with ACT and may have resulted in reducing population parasitaemia. Complementary surveys (e.g. WHO health facility surveys [26]) and analysis of longitudinal health facility data are being planned to assess whether observed decrease in malaria prevalence is true or in line with year-to-year epidemiological trends characteristic of malaria in Ethiopia [1].

While Ethiopia's scale-up in malaria interventions is a considerable achievement, the MIS data also shows some of the challenges the FMOH now faces in terms of malaria intervention coverage, use and/or access. First, early treatment-seeking behaviour remains far behind national or global targets and efforts to address this are urgently needed. With the recent training (including training in malaria treatment) and deployment of nearly 30,000 community-level health extension workers [27,28], Ethiopia is poised to address these low prompt treatment rates. Second, ITN household ownership is now high, but still short of the national and global 100% target, so work to achieve that target and then assure continued supply to maintain high ownership levels will be critical. Third, despite high ownership, ITN use remains lower than desired. The fact that 72% of households with an ITN had at least one person using the net the previous night is encouraging, but there is still room for improvement. A recent study of 15 standardized national surveys across Africa showed that within ITN-owning households, ITN use by children increases as the number of persons per available net decreases; notably of the 15 countries included in that study, Ethiopia was the one where this relationship did not hold true [29]. Thus, part of addressing ITN use will require assuring that households have adequate numbers of ITNs to be used by all household members, particularly children [30]. Exploration of other determinants of ITN use is critical [31] so that supporting interventions that would maximize ITN use (e.g. information, education, communication/behaviour, change, communication activities) can be designed and implemented.

As malaria prevalence decreases to the levels now seen in Ethiopia, malaria transmission is likely to be more and more focal, with some areas being relatively free and other areas still having substantial risk of malaria. Ethiopia now faces the challenge of further addressing this increasingly focal disease. This is not new in concept as Ethiopia has a long history of malaria epidemics. However, making further advances will require a proactive effort to further reduce transmission - in contrast to a reactive mode of waiting for outbreak detection and responding after they have occurred. Such proactive work would require Ethiopia to consider strengthening surveillance as an active intervention [32]. This might include strengthening malaria case management at community level through passive and active case detection by community health extension workers, in both remaining moderate transmission settings and in areas with very low transmission. With observed scale-up of malaria interventions since 2005, Ethiopia has shown that it can rise to the remaining challenges and ensure sustained malaria prevention and control efforts, and potentially embark on the path towards malaria elimination.

## Conclusions

Ethiopia's progress in malaria prevention and control demonstrates that with committed leadership, donor support, and strong partnership, African countries can produce remarkable results in scaling up anti-malaria interventions in a short time. This scale-up of malaria interventions is probably so far the largest of its kind in sub-Saharan Africa. However, the results of the MIS also show that activities supporting the scale-up and implementation of key malaria interventions need to occur to maximize the interventions' impact on disease morbidity and mortality. Sustaining the scale-up of malaria interventions is now of paramount importance for the Ethiopian FMOH and in-country malaria stakeholders, so that gains in terms of malaria prevention and control are not reversed and that malaria elimination becomes a truly achievable goal. With resources secured to support universal coverage of key malaria interventions by the end of 2010, Ethiopia is now poised to move from rapid scale-up for impact (SUFI) to sustained control, as a key step in the process towards eliminating malaria by 2020. The FMOH's 2011-2015 National Malaria Prevention and Control Strategy addresses these new challenges and focuses on vigorous community mobilization as the driving force to ensure continued provision of malaria prevention methods (LLINs and IRS), increased diagnosis and case detection, increased access to treatment, as well as a robust and active surveillance system to make sure its ambitious goals are attained.

## Competing interests

The authors declare that they have no competing interests.

## Authors' contributions

The Malaria Indicator Survey design, planning, implementation as well as the survey's data analysis and dissemination were the result of joint efforts by multiple individuals from malaria stakeholders inside and outside of Ethiopia. These individuals form the Ethiopia Malaria Indicator Survey Working Group and are listed as co-authors of this paper. HB and RR drafted the manuscript; JH led the analysis and presentation of the data for the manuscript; all authors provided extensive input into the review and finalization of the manuscript.

## Note

The Ethiopia Malaria Indicator Survey Working Group is comprised by: Mekonnen Amena, Laurent Bergeron, Hana Bilak, Brian Chirwa, Firew Demeke, Wubishet Dinkessa, Yeshewamebrat Ejigsemahu, Paul M Emerson, Tekola Endeshaw, Kebede Etana, Gashu Fente, Scott Filler, Anatoly Frolov, Khoti Gausi, Teshome Gebre, Tedros Adhanom Gebreyesus, Alemayehu Getachew, Asefaw Getachew, Patricia M Graves, Zelalem HaileGiorgis, Afework Hailemariam, Jimee Hwang, Daddi Jima, Henok Kebede, Abraham Lilay, Christopher Lungu, Ambachew Medhin, Addis Mekasha, John Miller, Aryc W Mosher, Hussein Muhamed, Sirgut Mulatu, Rory Nefdt, Jeremiah Ngondi, Dereje Olana, Richard Reithinger, Frank O Richards Jr, Amir Seid, Estifanos Biru Shargie, Richard Steketee, Zerihun Tadesse, Tesfaye Teferri, Agonafer Tekalegne, Eskindir Tenaw, Abate Tilahun, Adam Wolkon, Biratu Yigezu, Gedeon Yohannes
